# TUNEL labeling with BrdUTP/anti-BrdUTP greatly underestimates the level of sperm DNA fragmentation in semen evaluation

**DOI:** 10.1371/journal.pone.0181802

**Published:** 2017-08-07

**Authors:** Sofia C. Ribeiro, Monica Muratori, Maria De Geyter, Christian De Geyter

**Affiliations:** 1 Clinic of Gynecological Endocrinology and Reproductive Medicine, University Hospital, University of Basel, Basel, Switzerland; 2 University of Florence, Department of Experimental, Clinical and Biomedical Sciences-De Nothe Center of Excellence, Florence, Italy; University Hospital of Münster, GERMANY

## Abstract

Many studies have now confirmed that sperm DNA fragmentation (SDF) is associated with a poorer outcome of some forms of assisted reproduction technology. For this reason, SDF is an important parameter to evaluate in male fertility assessment. TUNEL (terminal deoxynucleotidyl transferase dUTP nick end labeling) assay coupled to flow cytometry is one of the most promising methods for SDF quantification. Several kits for the detection of DNA fragmentation are currently available on the market and all are recommended as equally appropriate to quantify SDF. In this work we compared for the first time the efficacy of two different types of TUNEL kits for SDF quantification: one using an indirect antibody-based labeling system (BrdUTP/fluorescein-anti-BrdUTP) and another using a direct labeling system (fluorescein-dUTP). We demonstrated that TUNEL indirect labeling system largely underestimates SDF when compared with the direct labeling, the differences ranging from 19.2% to 85.3% (p<0.05, n = 22). We observed that these differences were most pronounced among dead spermatozoa where indirect labeling stained 40.1% [23.6%, 58.2%] and the direct system 65.7% [36.5%, 90.9%] (n = 10, p<0.05). Interestingly, we found that both systems stained the living spermatozoa with the same efficiency. We showed that the differences are due to the steric hindrance of the antibody during its binding to the BrdUTP. Indeed, after sperm DNA decondensation, the percentages of TUNEL positivity increased significantly from 46.3% [31.8%, 61.7%] to 97.5% [96.1%, 98.8%] (p<0.05, n = 5). Our results are important for future use of TUNEL in clinical practice. Laboratories relying on the use of an antibody-based system heavily underestimate SDF, most particularly in infertile patients with reduced sperm motility. As a consequence, the kit using BrdUTP/fluorescein-anti-BrdUTP should not be recommended as a method to assay DNA damage in semen. This study represents one further step in the standardization of TUNEL among laboratories.

## Introduction

Ever since the publication of the first reports [1, 2] the role of sperm DNA fragmentation (SDF) in understanding male infertility has steadily grown. Meanwhile, numerous studies have confirmed its adverse effects on reproductive outcome [3–11]. Despite this progress medical societies such as the American Society for Reproductive Medicine [12], the European Society for Human Reproduction and Embryology [13] and the British Fertility Society [14] still argue that the true value of SDF still needs to be established. In consequence, SDF was not adopted into the latest WHO guidelines [15]. The exact technology to be used for the quantification of SDF and the lack of standardization of each of the proposed methods remain controversial.

Terminal deoxynucleotidyl transferase dUTP Nick End Labeling (TUNEL) ranks among the most promising assays for the quantification of SDF. TUNEL measures both single and double DNA-strand breaks through the enzymatic incorporation of a modified dUTP at the 3’-OH terminal end of a DNA strand. As such, TUNEL directly quantifies DNA damage, which in comparison to all other methods, constitutes one of its advantages [16]. The modified dUTP can be labeled either directly (with fluorescein-dUTP) or indirectly through the use of labeled antibodies or streptavidin (the latter for biotin-dUTP). Other widely used tests such as the Sperm Chromatin Structure Assay (SCSA) [17], Sperm Chromatin Dispersion (SCD) [18] or alkaline Comet assay [19], which assess the chromatin structural integrity of spermatozoa by testing its susceptibility to acid or alkaline -induced denaturation and thereby assess DNA damage indirectly.

Quantification with TUNEL can be carried out with flow cytometry (TUNEL-FC) which allows the rapid assessment of more than 10,000 spermatozoa per sample. Current protocols combine TUNEL with the staining of the nucleus with propidium iodide (PI) to exclude apoptotic bodies in the semen [20], which interfere with the TUNEL analysis [21–23]. In addition, TUNEL-FC allows the discrimination of spermatozoa with two distinct PI-fluorescence intensities (brighter and dimmer populations) [20, 24] each with different clinical correlations [6, 25] and biological properties [25]. The PI-dimmer reflects dead spermatozoa whereas the PI-brighter population consists of spermatozoa with variable fractions of both living and dead spermatozoa [20]. Staining with PI adds to the diagnostic power of flow cytometry and TUNEL as SDF in the brighter population predicts male fertility independently from conventional semen parameters and male age. Particularly at high SDF, this subpopulation discriminates better between infertile patients and fertile controls than SDF measured in the total sperm population [6].

The potential of TUNEL-FC combined with PI-staining makes this technique a promising candidate to become the golden standard in quantification of SDF in semen. However, in different laboratories the threshold values correlating with male infertility fluctuate between 20% [26, 27] and 35% [28, 29] indicating that we are still far from an established and standardized method.

Although several kits for the detection of DNA fragmentation are currently available on the market none was originally developed for semen analysis. Despite this, all are recommended as equally appropriate to quantify the DNA damage in sperm [30–32]. Among the available systems the BrdUTP/anti-BrdUTP TUNEL labeling is based on the incorporation of 5-Bromo-2'-deoxyuridine-5'-triphosphate (BrdUTP) in the DNA extremities. BrdUTP is subsequently detected with an anti-BrdUTP antibody covalently bound to fluorescein (FITC-anti-BrdUTP). This method was originally described as being the most sensitive and the one giving rise to brighter TUNEL signals in FC because of the rapid incorporation of BrdUTP into the DNA of fragmented cells due to minimal steric hindrance [33, 34].

In this study we compared the BrdUTP /FITC-anti-BrdUTP TUNEL labeling system with the direct staining with fluorescein-dUTP for detection of DNA fragmentation in spermatozoa. This is the first study that directly compares different labeling systems in TUNEL for semen analysis.

## Materials and methods

### Semen samples

This study was part of a comprehensive and prospective validation of the TUNEL assay as carried out in the Clinic of Gynecological Endocrinology and Reproductive Medicine, University Hospital, Basel, Switzerland. The validation process was presented to the review board of the ethics committee of Northwestern & Central Switzerland (EKNZ). The board decided that the procedures outlined here were part of ongoing quality assurance and as such did not require a formal approval. The TUNEL-FC validation process involved the analysis of 32 semen samples of both normozoospermic and non-normozoospermic men (according to the 2010 WHO reference values) attending the Clinic between July 2015 to March 2017 (S1 Table). The selection of the semen samples was random and based only on the criterion of containing a sufficient number of spermatozoa necessary for the controls and analysis for each assay. Samples with a very low total sperm number (less than 16 million) were, for this reason, excluded. Subjects collected samples by masturbation after 2 to 7 days of sexual abstinence according to the WHO-guidelines.

### Conventional semen analysis

Samples were collected in 110 ml sterile plastic containers (BD Falcon, Franklin Lakes, NJ, USA) and allowed to liquefy at 37°C for 30 minutes. Conventional semen analysis was carried out semi-automatically using Sperm Computer Analysis (SCA, Microptic, Barcelona, Spain) in all samples and the cut off values were given by the WHO-guidelines (2010) [15].

### TUNEL assay

#### TUNEL protocol

In each semen sample the TUNEL assay was performed simultaneously with the *Apo-BrdU In Situ DNA-Fragmentation* kit (BioVision, Milpitas, CA, USA, from here indicated as indirect kit) and the *In Situ Cell Death Detection*, *Fluorescein* kit (Roche Diagnostics, Rotkreuz, Switzerland, from here indicated as direct kit) according to manufacturer’s instructions and protocols developed specifically for the analysis of spermatozoa [20]. Briefly, 2 x 10^6^ spermatozoa were centrifuged at 400 g for 7 minutes and resuspended in PBS containing 3.7% of paraformaldehyde (PFA, Thermo Scientific, Rockford, IL, USA) for 15 minutes. After fixation, the samples were centrifuged at 400 g for 7 minutes and resuspended in a permeability enhancing solution containing Triton 0.1% (Sigma, St Louis, MO, USA) in 0.1% sodium citrate (Sigma, St Louis, MO, USA) for 2 min at 4°C. Then the cells were washed twice with the washing solution provided in the indirect kit or PBS 1x in the case of samples processed with the direct kit, and incubated for one hour at 37°C in the DNA labeling solution containing the reaction buffer, the terminal deoxynucleotidyl transferase (TdT) enzyme and the BrdUTP or FITC-dUTP supplied with the indirect or direct kits, respectively. One negative control, which contained all the components of the labeling solution except the enzyme TdT, was always included.

The samples were then washed twice with the rinse buffer provided in the indirect kit or PBS 1x in the case of direct assay. The latter samples were incubated in 500 μl of PBS 1x containing 10 μl of PI (Molecular Probes, Eugene, OR, USA) 30 μg/ml for 30 minutes in the dark and then analyzed with BD Accuri C6 flow cytometer (BD Biosciences, San Jose, CA, USA).

Samples processed by indirect kit were further incubated in 100 μl of the FITC-anti-BrdUTP antibody solution for 30 minutes at room temperature in the dark. Finally, PI/RNase was added and after 30 minutes of incubation at room temperature in the dark the cells were analyzed with BD Accuri C6 flow cytometer. Since RNase treatments was not present in samples processed with the direct kit, in some experiments RNase was omitted in the PI solution. Results indicated that the treatment with the enzyme did not affect the measures of SDF with the indirect kit. Duplicates were always performed for each sample. TUNEL-positive cells were identified as PI-positive (FL2-channel, 585/40 nm filter) and FITC-positive cells, (FL1-channel, 530/40 nm filter). During data analysis of TUNEL/PI labelled sperm samples, a 2% value for compensation of FITC fluorescence spillover on PI channel was used as determined in a TUNEL labelled sperm sample where PI staining was omitted. When necessary, for comparison reasons, a fluorescein isothiocyanate (FITC) labeled dUTP from BD Pharmigen (BD Biosciences, San Diego, CA, USA) was also used in the TUNEL assay. In this case the same protocol was followed as described for the indirect kit but replacing the BrdUTP for FITC-dUTP and omitting the incubation with FITC-anti-BrdUTP.

#### TUNEL-FC data analysis

**Fig 1:**
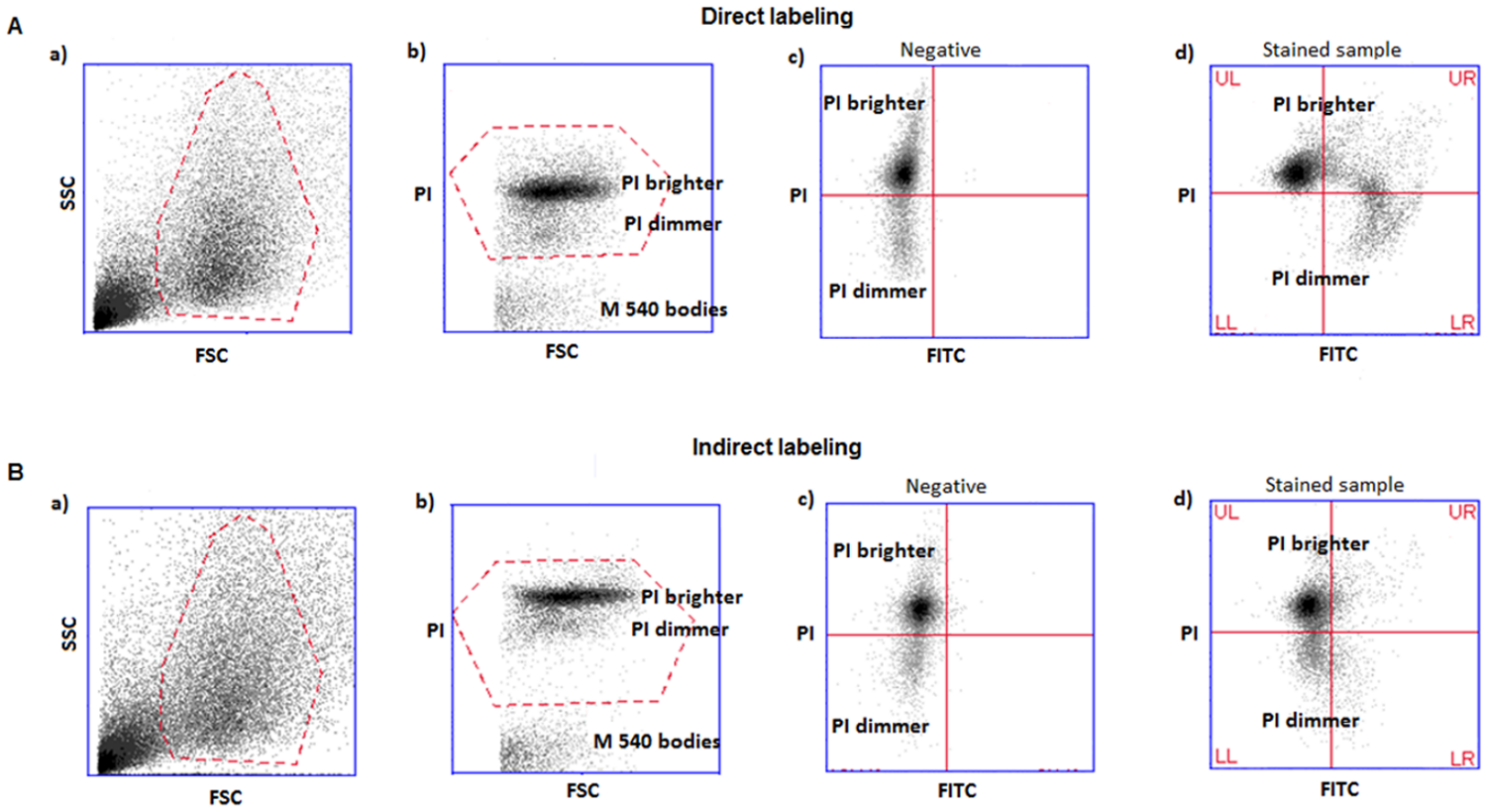
Example of dot plots obtained and strategy followed in TUNEL-FC analysis. **A**) direct labeling and B) indirect labeling. a) FSC/SSC plots and flame shaped region (red) including spermatozoa and semen apoptotic bodies; b) PI/FSC plots and region (red) including PI-positive events (i.e. all spermatozoa) and excluding M540 bodies. Note the presence of two sperm populations with different PI staining intensity (PI brighter and PI dimmer); c) PI/FITC plots of negative control (without TdT enzyme), where quadrants are set; d) PI/FITC plots of a test sample, where TUNEL-positivity is calculated. Percentage of spermatozoa in UR quadrants reveals TUNEL-positivity of the PI-brighter population. Percentage of spermatozoa in LR quadrants reveals TUNEL-positivity of the PI-dimmer population. FSC-forward scatter, SSC-side scatter, PI- propidium iodide fluorescence, FITC-fluorescein isothiocyanate fluorescence, UL-upper left quadrant, UR-upper right quadrant, LL-lower left quadrant, LR-lower right quadrant. PI-dimmer and PI-brighter populations are indicated.

TUNEL/PI dot plots and the strategy used for the acquisition of the TUNEL data are shown in Fig 1. In panel a, we report the FSC (forward scatter)/SSC (side scatter) dot plots in which spermatozoa are gated in a flame shape region, which contains the spermatozoa and excludes debris and larger cells. In the panel b (PI fluorescence/FSC), spermatozoa are selected by gating the PI-positive cells. PI is a DNA intercalating fluorescent dye that enters only to dead cells (cells with permeable membranes). Since all spermatozoa were previously fixed and permeabilized, PI will enter into all spermatozoa present in the sample but will not stain semen apoptotic bodies that lack chromatin and that are contained in the same flame shape region as spermatozoa [35]. Finally, the percentages of TUNEL-positive spermatozoa are obtained in the PI/FITC plots (c: negative control; d: stained samples) as a percentage of spermatozoa that are positive both for PI and FITC (the sum of upper right, UR, and lower right, LR, quadrants). The negative control dot plots were used to set the quadrants for sample analysis.

Although all spermatozoa are dead after the TUNEL assay, two distinct populations can be observed with PI staining in the cytometry data analysis: a population staining less intensively with PI (PI-dimmer) and a population staining with a brighter PI-fluorescence intensity (PI-brighter) (visible in plots b, c and d). The use of quadrants in the data analysis allows the separate quantification of TUNEL-positivity in PI-dimmer and PI-brighter populations.

### TUNEL- LIVE/DEAD assay

#### TUNEL- LIVE/DEAD staining procedure

Since after TUNEL procedure all the spermatozoa are dead, it is not possible to distinguish spermatozoa that were dead from those that were alive in the freshly collected ejaculate. In order to be able to distinguish SDF between dead and living spermatozoa, 10 semen samples were simultaneously labeled with the LIVE/DEAD Fixable Far Red-kit (LIVE/DEAD dye) (Molecular Probes, Eugene, OR, USA) and TUNEL. Each sample was measured in duplicate. The semen aliquots were centrifuged at 400 g for 7 minutes and the pellet resuspended in 1 ml PBS 1x containing 0.5% of human serum albumin (HSA) (Sigma-Aldrich, St Louis, MO, USA) and 1 μl of the LIVE/DEAD dye. After 30 min of incubation at 37°C, the cells were centrifuged at 400 g for 7 minutes, fixed with PFA 3.7%, processed with both TUNEL kits and finally stained with PI as described above.

#### TUNEL- LIVE/DEAD data analysis: SDF in dead and live spermatozoa

**Fig 2:**
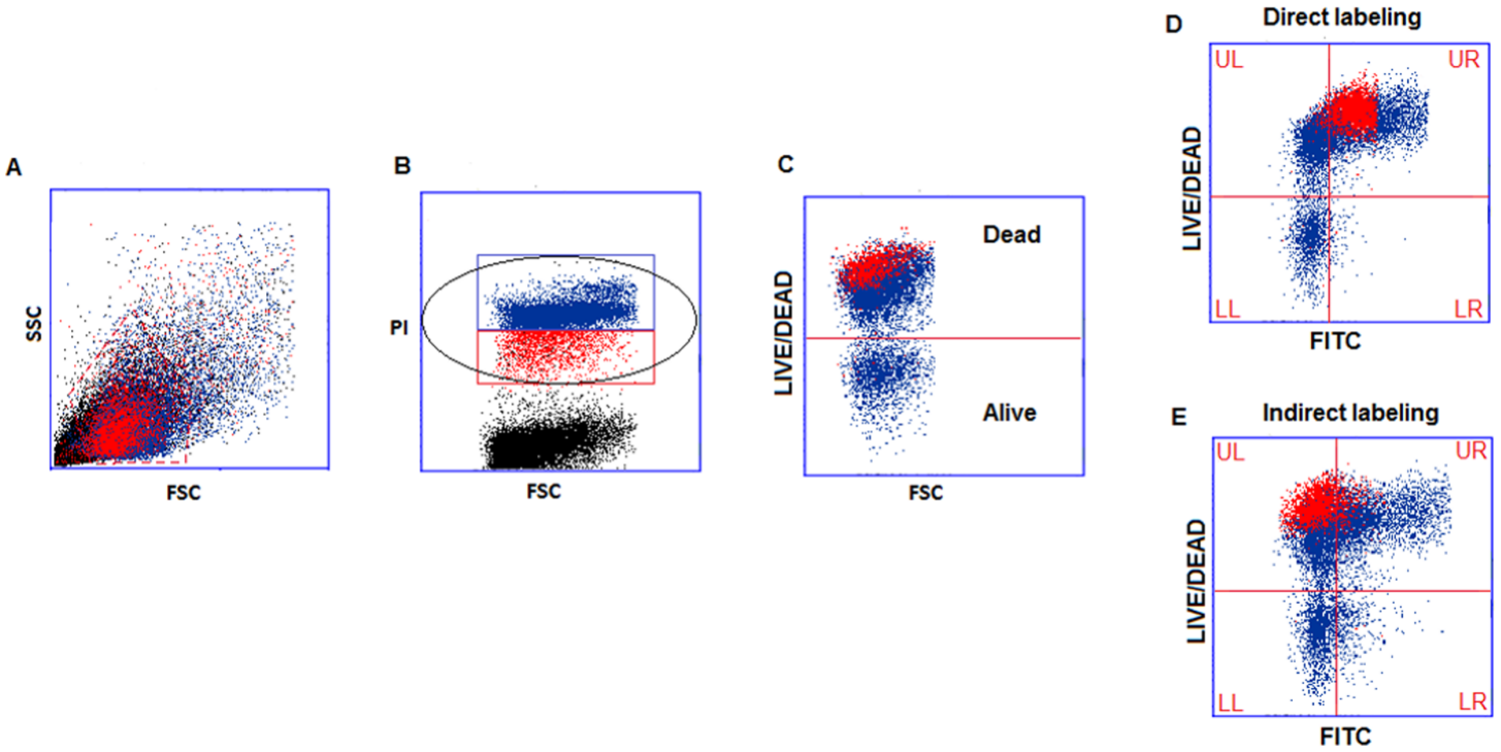
TUNEL-LIVE/DEAD data analysis. A) FSC/SSC plots and flame shaped region containing spermatozoa and semen apoptotic bodies. B) PI/FSC plots showing the gates for selection of all spermatozoa (black ellipse) or PI-brighter (blue) and PI-dimmer (red) populations. C) DEAD/ALIVE/FSC plot with the dead cells showing positive staining for LIVE/DEAD dye (upper region). D) and E) LIVE/DEAD/ FITC plots for direct and indirect staining, respectively. The UR quadrants show dead spermatozoa staining for TUNEL and LR quadrants show live spermatozoa staining for TUNEL. By selecting, in plot B, only the PI-brighter population (in blue) or the PI-dimmer population (in red) the Accuri C6 software can quantify separately the SDF in the distinct dead populations. UL-upper left quadrant, UR-upper right quadrant, LL-lower left quadrant, LR-lower right quadrant.

TUNEL- LIVE/DEAD dot plots and the strategy used for the quantification of SDF in dead and living spermatozoa are shown in Fig 2. The LIVE/DEAD dye permeates the damaged membranes of dead spermatozoa and reacts with free amines both in the interior and on the surface, yielding intense fluorescent staining (Fig 2C upper region). In living spermatozoa, the dye reacts only with the surface amines, resulting in less intense fluorescence (Fig 2C, lower region). Unlike PI dye, LIVE/DEAD dye remains inside the dead cells even after the fixation and permeabilization steps of the subsequent TUNEL protocol (Fig 2D and 2E).

As in TUNEL-FC analysis, PI-positive elements were selected (Fig 2B, black ellipse). All spermatozoa are stained with PI, but only those that were dead in the fresh ejaculate will be stained with LIVE/DEAD dye (Fig 2D and 2E, upper region). Finally, the simultaneous analysis of the LIVE/DEAD dye fluorescence and FITC fluorescence allows determining DNA fragmentation among dead and living spermatozoa separately (Fig 2E and 2D, upper right and lower right quadrants, respectively).

#### TUNEL-LIVE/DEAD data analysis: SDF in distinct dead populations (dead PI-brighter and dead PI-dimmer)

By selecting in PI/FSC panel (Fig 2B) only the PI-brighter or the PI-dimmer populations (Fig 2B, blue or red rectangles, respectively), the Accuri C6 software can quantify SDF in spermatozoa belonging to each population. Using the software colored gates it was clearly observed that PI-brighter population (Fig 2D and 2E in blue) is composed of dead and living spermatozoa while PI-dimmer (Fig 2D and 2E in red) is composed of only dead spermatozoa. The dead PI-brighter or the dead PI-dimmer populations were then identified as the blue or red populations, respectively, in the upper quadrants of the LIVE/DEAD/FITC panels (Fig 2D and 2E upper left + upper right quadrants). The SDF is calculated as the percentage of blue or red population present in the upper right quadrants. These values were determined for the 10 semen samples processed with TUNEL- LIVE/DEAD assay.

### Sorting of PI-brighter and PI-dimmer spermatozoa

Aliquots of freshly collected ejaculates (2 to 5 x 10^6^ spermatozoa) from 5 patients were mixed with 1 ml of Sydney IVF Sperm Medium (COOK medical, Brisbane, Australia) and centrifuged for 7 minutes at 400 g. Then, the pellets were suspended in 1 ml of PBS 1x with 20 μl of PI 30 μg/ml for 30 minutes. Before sorting, the stained samples were filtered with CellTrics 100 μm-filters (Partec, Görlitz, Germany). PI-dimmer or PI-brighter populations were isolated using the BD Influx cell sorter (BD Biosciences, San Jose, CA, USA) and the Sortware software (BD Biosciences, San Jose, USA). The following settings were used for sorting: 80 μm nozzle, sample pressure: 14–15 psi, event rate: 3,000–4,000 events/second. Sterile PBS 1x solution was used as sheath fluid and the sorted spermatozoa were collected in 14 ml polystyrene round-bottom tubes (BD Falcon tubes, Biosciences, Durham, NC, USA), containing 1 ml of the Sydney IVF-medium. The sorting was aborted after 1x10^6^ spermatozoa were obtained. The sorted samples were centrifuged at 400 g for 7 minutes and resuspended in 1 ml Sydney IVF Sperm Medium.

### Chromatin decondensation in PI-brighter and PI-dimmer spermatozoa

For the decondensation of sperm chromatin we adopted a protocol published elsewhere [36]. Briefly, aliquots from sorted populations containing approximately 1 x 10^6^ spermatozoa were washed with 4 ml of PBS 1x/0.5% HSA and chromatin stabilization was performed by incubation in 500 μl of 0.5% PFA at 4°C for 10 minutes. Samples were then centrifuged and resuspended in the decondensation solution (PBS 1x containing 5 mM dithiothreitol (DTT, Carl Roth, Karlsruhe, Germany); 0.1% Triton and 100U/ml of low molecular weight heparin (Fragmin, Pfizer, New York, NY, USA) or in PBS containing 0.5% HSA in case of the non decondensed samples. During decondensation (30 minutes in the dark at 25°C), every 10 minutes, spermatozoa were counted under the microscope to exclude loss of cells. Microscopic analysis revealed that the number and the integrity of the PI-dimmer and PI-brighter spermatozoa were maintained during the process. Finally the samples were centrifuged and fixed in PFA 3.7% overnight at 4°C. To verify whether the sperm DNA was efficiently decondensed, aliquots of decondensed and non-decondensed samples were re-stained with PI and flow cytometry analysis was performed. A significant increase in the PI-fluorescence of the decondensed population was observed in all cases. These samples were subsequently used to test the influence of chromatin decondensation in TUNEL results.

### Statistical analyses

Differences between the not-normally distributed populations were assessed with Wilcoxon Signed Rank test and One-Way ANOVA was used for smaller samples sizes (n = 5). Correlations significance was tested with Spearman's rank correlation coefficient. Data were expressed as mean % together with the 95% confidence interval. The level of statistical significance was set at the 5% level.

## Results

### Comparison between TUNEL results obtained with indirect (BrdUTP/FITC-anti-BrdUTP) and direct (FITC-dUTP) labeling

TUNEL-FC results are given as the subpopulation of spermatozoa (in %) that simultaneously stain positive for PI and for the fluorophore used to label the DNA strand breaks (Fig 1). In all 22 semen samples, two populations with distinct degrees of PI fluorescence were visible: one population staining more intensively (PI-brighter) and another population staining less intensively with PI (PI-dimmer) (Fig 1, panels b, c and d).

**Fig 3:**
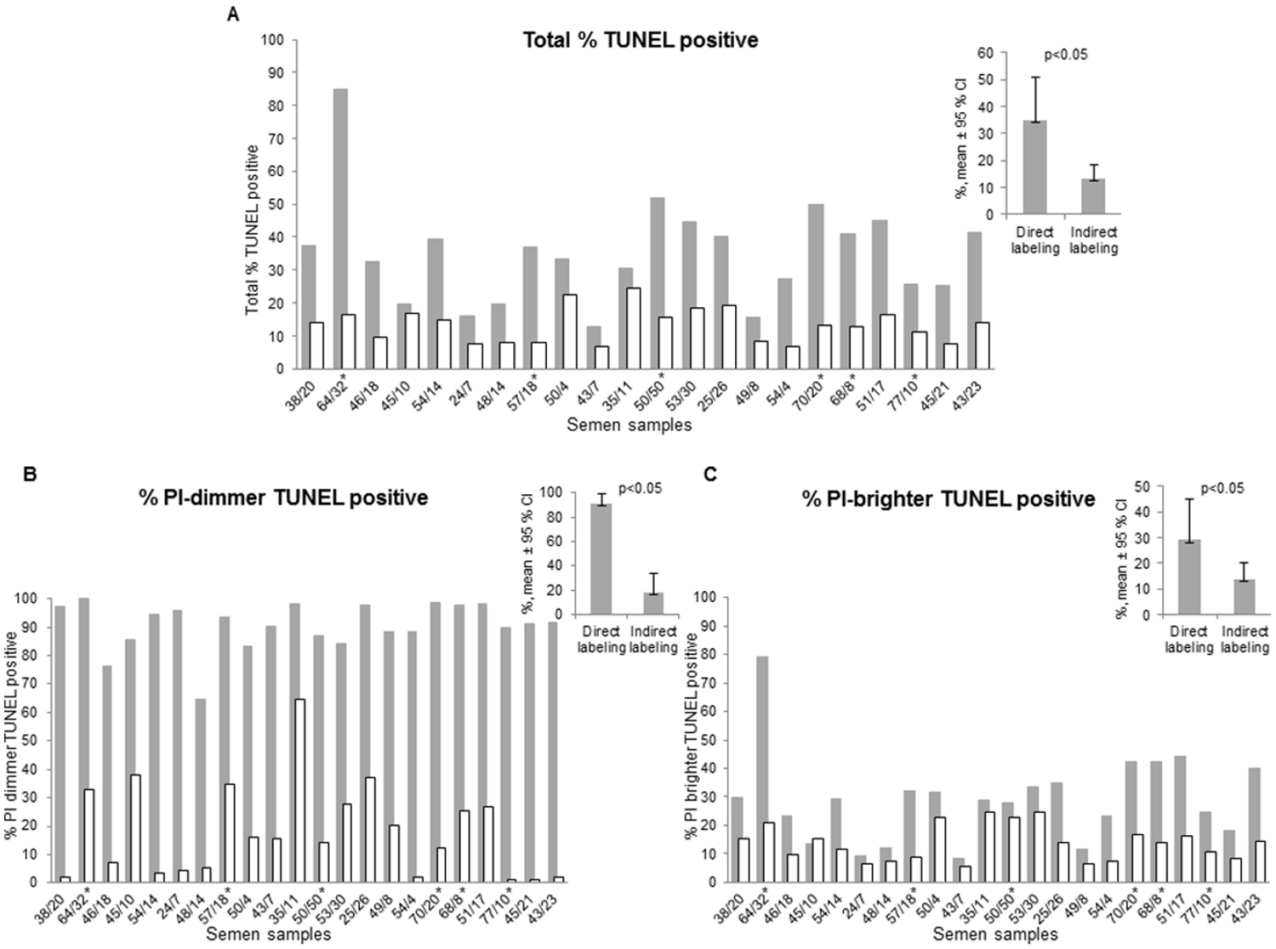
Comparison of TUNEL results obtained with the two kits. (A) Total percentage of TUNEL-positive spermatozoa; (B) Percentage of PI-dimmer population positive for TUNEL; (C) Percentage of PI-brighter population positive for TUNEL. Direct labeling: grey columns; Indirect labeling: white columns. For each semen sample the percentage of immotile spermatozoa calculated in the conventional semen analysis and the percentage of PI-dimmer population obtained after PI-staining during TUNEL assay are indicated in the X-axis, in the first sample, 38/20 corresponds to a sample with 38% of immotile spermatozoa and 20% of PI dimmer spermatozoa. The non-normozoospermic men (2010 WHO reference values) are indicated with an asterisk (S1 Table). CI-confidence interval, p-Wilcoxon Signed Rank p.

When stained with indirect labeling (BrdUTP/FITC-anti-BrdUTP) the percentage of TUNEL-positive cells was invariably lower, the values ranging from 19.2% to 85.3% of the percentage of TUNEL-positive cells stained by direct labeling using fluorescein-dUTP (p<0.05, n = 22) (Fig 3A). These differences were more pronounced in semen samples with lower quality, as characterized by total motility below 40%, or a percentage of PI-dimmer spermatozoa higher than 14% (Fig 3, x-axis legend). We found a statistically significant correlation between the percentage of immotile spermatozoa and the magnitude of the difference between the kits: the larger the number of immotile spermatozoa the larger is the difference (P<0.05). The same correlation was observed for the percentage of PI-dimmer population. No correlation was found between the magnitude of the differences and the semen volume, concentration or percentage of normal forms.

The semen characteristics of each sample are shown in S1 Table. The order of the samples in Fig 3 is the same as the one presented in S1 Table.

The differences between the two labeling systems were even more pronounced when only the PI-dimmer populations were examined. With direct TUNEL-labeling virtually all PI-dimmer spermatozoa were stained (LR quadrants of panels of Fig 1A and 1B plots d and Fig 3B), whereas with the indirect TUNEL-labeling this population was stained to a much lesser extent (LR quadrants of dot plots of Fig 1b and 1d). Indirect labeling stained 17.9% of the PI-dimmer population [11.0%, 24.8%], whereas with direct labeling 90.6% of the PI dimmer population was stained [87.1%, 94.1%] (Fig 3B).

In the PI-brighter population (Fig 3C), the observed differences between both TUNEL staining methods were less pronounced, although still reaching statistical significance (p<0.05). The number of spermatozoa stained with indirect TUNEL-labeling generally amounted to only 30 to 100% of the number of spermatozoa stained with the direct TUNEL-labeling (Fig 3C).

### Testing the kits enzymes and labeling systems

**Fig 4:**
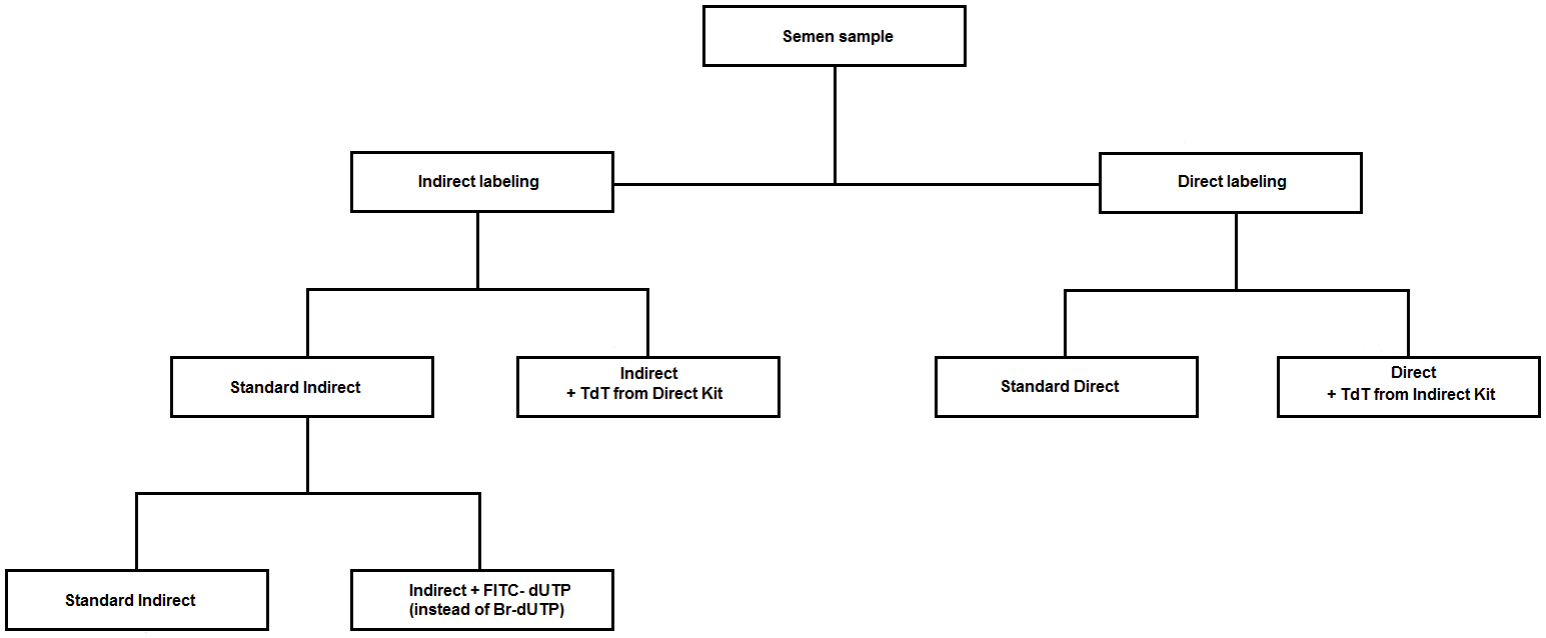
Flow chart showing the strategy of exchange of kits components.

To evaluate whether the observed differences in staining efficiencies were caused by a different TdT-enzyme activity, semen samples (n = 5) were labeled with: 1) standard indirect labeling; 2) indirect labeling using the enzyme TdT provided by the direct labeling kit (indirect/direct TdT); 3) standard direct labeling; 4) direct labeling using the enzyme TdT provided by the indirect labeling kit (direct kit/indirect TdT) (Fig 4).

TUNEL performed with standard indirect labeling or with indirect labeling/direct TdT yielded similar percentages of fragmented spermatozoa in PI-dimmer populations, respectively: 14.1% [5.4%, 22.8%] and 14.7% [6.1%, 23.3%] (n = 5, p>0.05). Also standard direct labeling and direct labeling/indirect TdT gave similar results, respectively: 85.3% [75.7%, 94.8%] and 75.4% [62.5%, 88.1%] (n = 5, p>0.05). However, the percentages obtained with indirect labeling and indirect labeling/direct TdT were significantly lower (p<0.05) than those obtained with direct labeling and direct labeling/indirect TdT (p>0.05). This finding revealed that the enzymes are equally efficient, as they yielded similar staining intensities either with indirect labeling or with direct labeling. In addition, the reduced staining with the indirect system, irrespective of the enzyme used, suggest that other components of this kit were responsible for the poorer labeling efficiency. To confirm this, we used all the indirect labeling components, but replaced the indirect labeling system (BrdUTP/FITC-anti BrdUTP) with a direct one (FITC-dUTP). In this set of experiments (n = 5), we observed an increase of the PI-dimmer staining rates from 22.7% [11.3%, 34%] to 75.5% [66.2%, 84.8%], indicating that the labeling system itself was responsible for the observed differences, not the other components of the kits. In the indirect system an antibody was used for labeling, which is a considerably larger molecule than the FITC-nucleotide, used in the direct system. We therefore hypothesized that the differences in the staining efficiency of both kits was caused by the steric hindrance of the antibody during its binding to the BrdUTP.

### Testing of the steric hindrance: Effect of spermatozoa chromatin decondensation on TUNEL results

**Fig 5:**
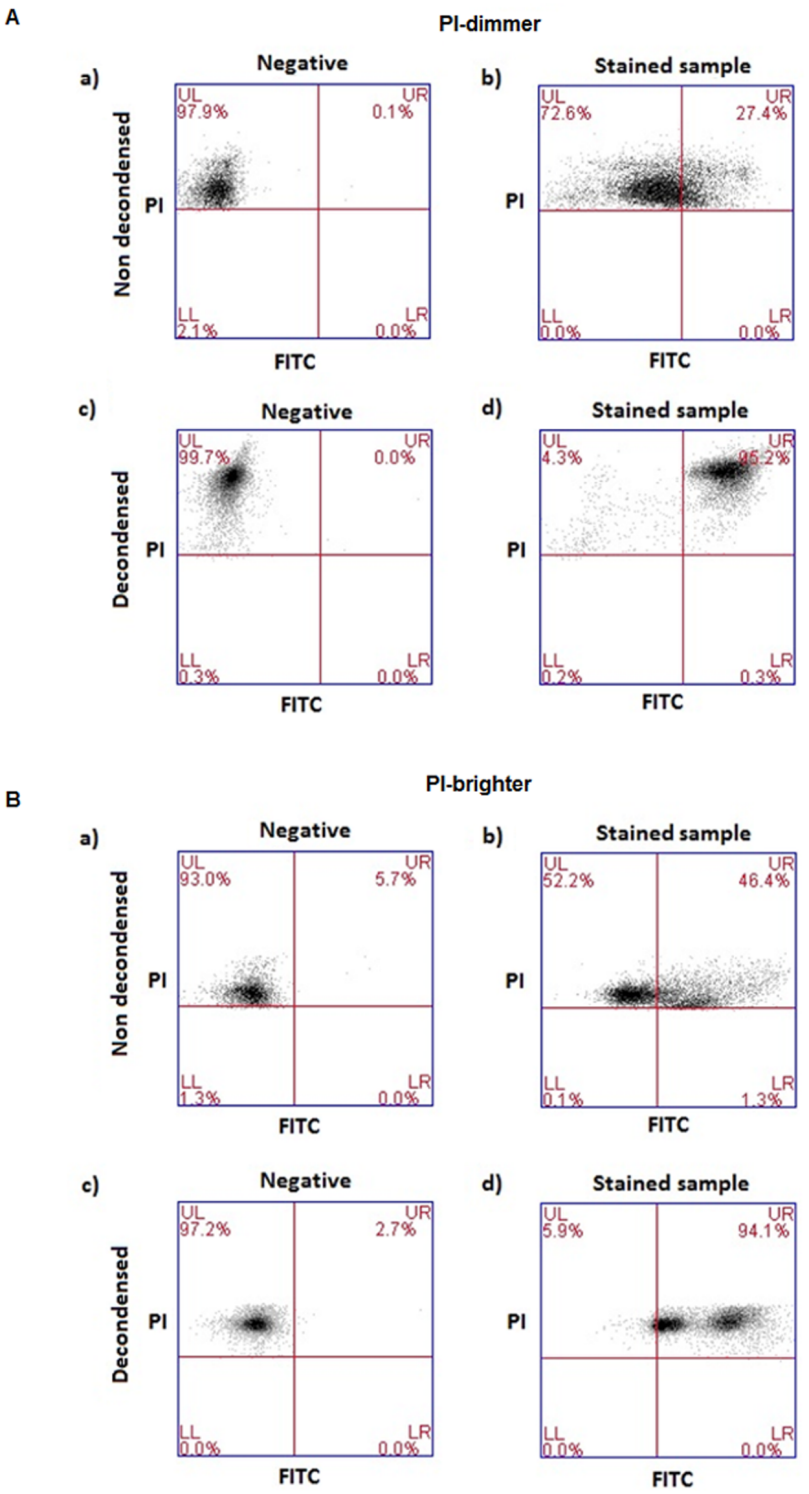
Example of dot plots obtained in TUNEL analysis with the indirect labeling system after decondensation of spermatozoa populations. A) PI-dimmer; B)-PI- brighter. a) negative controls of non decondensed samples, b) stained non decondensed samples, c) negative controls of decondensed samples, d) stained decondensed samples. PI- propidium iodide fluorescence, FITC-fluorescein isothiocyanate fluorescence; UL-upper left quadrant, UR-upper right quadrant, LL-lower left quadrant, LR-lower right quadrant.

To verify whether steric hindrance of the anti-BrdUTP labeling molecule to the compact DNA of spermatozoa caused the lower efficiency of the indirect labeling method, we performed a three step experimental analysis using five freshly collected semen samples. We first stained the dead spermatozoa in semen samples with PI and then sorted the populations into PI-dimmer and PI-brighter populations. We then decondensed the chromatin of each sorted population, keeping an aliquot of each sorted sample untreated (undecondensed). Finally, with TUNEL we quantified the SDF using the indirect labeling in all 4 fractions (Fig 5).

Surprisingly, decondensation of the chromatin of spermatozoa from the PI-dimmer fraction raised the level of PI-fluorescence to the level of the decondensed PI-brighter population. In addition, after decondensation, the percentages of TUNEL positivity in PI-dimmer sperm increased significantly from 46.3% [31.8%, 61.7%] to 97.5% [96.1%, 98.8%] (p<0.05, n = 5) and in the PI-brighter population from 57.1% [44%, 70.2%] to 87% [74.3%, 100.0%] (p<0.05, n = 5) (Fig 5).

### Comparison between TUNEL staining efficiencies in individual spermatozoa populations

#### Live and dead spermatozoa

**Fig 6:**
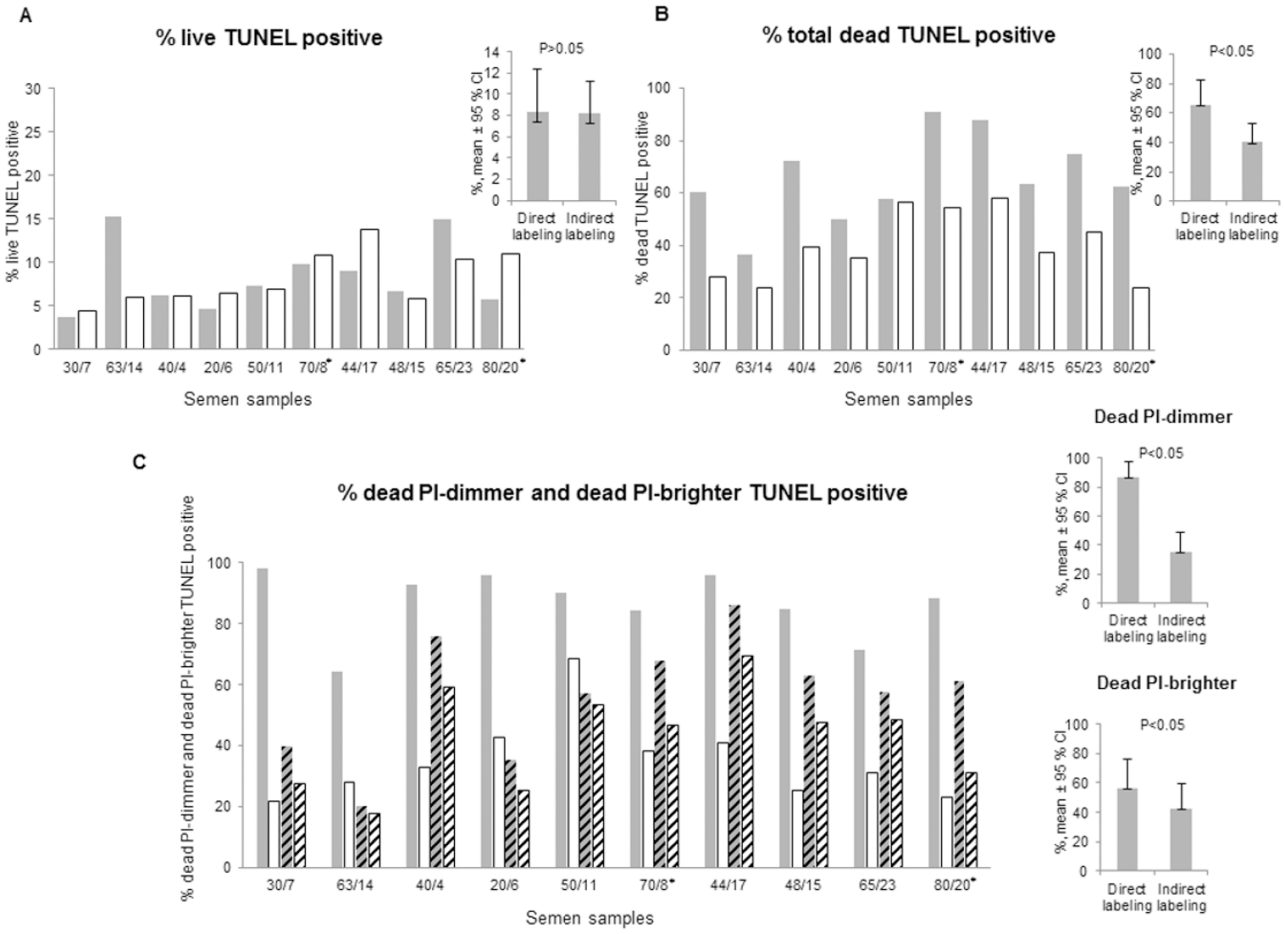
Comparison of TUNEL-LIVE/DEAD results. (A) Percentage of live population positive for TUNEL; (B) Percentage of total dead population positive for TUNEL; (C) Percentage of dead PI-dimmer and dead PI-brighter populations positive for TUNEL. Direct labeling: filled or stripped grey columns. Indirect labeling: filled or stripped white columns. For each semen sample the total percentage of dead spermatozoa stained with LIVE/DEAD kit and the percentage of PI-dimmer population are indicated in the X-axis. The non-normozoospermic men (2010 WHO reference values) are indicated with an asterisk. CI-confidence interval, p- Wilcoxon Signed Rank p.

The lower percentages of TUNEL-labeled cells obtained with the indirect labeling method as compared to the direct labeling method were observed mainly in the PI-dimmer population, although some differences between both TUNEL-labeling methods were observed in the PI-brighter population as well (Fig 3C). PI-brighter and PI-dimmer populations differ on the content of dead cells that are present in variable amounts in the former, whereas in the latter the entire population of spermatozoa consists of dead cells [20]. Given that, we hypothesized that the differences in the staining between the two systems were caused by the condensed chromatin architecture in dead spermatozoa. In order to distinguish between TUNEL results in living and dead populations we performed a staining with the LIVE/DEAD Fixable Far Red kit before carrying out the TUNEL assay (TUNEL-LIVE/DEAD) (Fig 2). The semen characteristics of each sample used in TUNEL-LIVE/DEAD assay are shown in S1 Table. The order of the samples in Fig 6 is the same as the one presented in S1 Table.

The LIVE/DEAD stain enters only into dead spermatozoa, whereas living populations remain unstained (Fig 2B). After the TUNEL assay it was possible to separately quantify the dead TUNEL-positive spermatozoa (Fig 2D and 2E, UR quadrants) and the fraction of the living TUNEL-positive spermatozoa (Fig 2D and 2E, LR quadrants). No significant differences between the percentages of living spermatozoa stained with either TUNEL labeling method were observed (Fig 6A, direct labeling: 8.3% [5.8%, 10.8%], indirect labeling: 8.9% [6.3%, 10.1%] (n = 10, p>0.05).

As expected, the staining efficiency of the dead population is significantly lower with the indirect labeling 40.1% [23.6%, 58.2] (n = 10, p<0.05), when compared with the direct 65.7% [36.5%, 90.9%] (n = 10, p<0.05), since it includes the PI-dimmer subpopulation of spermatozoa (Fig 6B).

#### Distinct dead spermatozoa populations: Dead PI-brighter and dead PI-dimmer

Using the Accuri C6 cytometer software and by selecting the PI-dimmer or the PI-brighter populations (Fig 2B), the percentage of TUNEL-positive spermatozoa among the dead spermatozoa in each population, i.e. dead PI-brighter and dead PI-dimmer, were quantified (Fig 2D and 2E, respectively). Using the indirect labeling the staining intensity of the PI-dimmer population was much lower than with the direct labeling (n = 10, p<0.05) (Fig 6C). In the dead PI-brighter populations the differences in the staining intensities of both kits were smaller but still statistically significant (p< 0.05) (Fig 6C).

## Discussion

Among all existing quantification methods of SDF TUNEL seems to be one of the most promising, as it directly examines the condition of the DNA [37]. This assay has the advantage, shared with SCSA, to be detected by FC enabling the fast assessment of many spermatozoa. However, existing protocols must be standardized better and validated before they can be adopted in prospective studies or in clinical routine.

Current TUNEL technology for the quantification of spermatozoa with single or double DNA strand breaks relies on commercially available kits. In principle, any spermatozoon with damaged DNA should be stained efficiently regardless the commercial kit used. A comprehensive list of commercially available kits using either direct or indirect systems is presented in S2 Table. In the present study we demonstrate that those TUNEL kits which detect DNA strand breaks using a fluorescent antibody (BrdUTP/FITC-anti-BrdUTP) largely underestimate SDF, if compared to a direct labeling system such as fluorescein-dUTP.

Here, we found remarkable differences in the results of TUNEL among the PI-dimmer population (Fig 3B), when TUNEL was performed either with direct labeling or when an antibody-based system was used to reveal dUTPs. In contrast to PI-dimmer populations, this difference was smaller but still significant in PI-brighter populations. The PI-dimmer population (denominated as such because of the weaker staining with the nuclear dye PI) is known to consist of spermatozoa that were already dead in the freshly collected semen sample prior to fixation for TUNEL [38]. The PI-brighter population, strongly staining with PI, consists of spermatozoa with variable fractions of both living and dead spermatozoa [20].

After having investigated the mechanisms involved in causing these differences more in detail, we hypothesized that the steric hindrance of the antibody to the access into sperm chromatin may have been responsible for the lower staining intensity when using the indirect labeling system. Indeed, when the TdT enzyme provided by the indirect labeling kit was substituted by the same enzyme provided by the direct labeling kit, TUNEL staining intensity did not increase, indicating that the cause for the lower values obtained with this system was not the enzyme. Similar conclusions were drawn from the results of experiments conducted with direct labeling but using the TdT of the indirect labeling kit. Conversely, when in the indirect labeling procedure, the BrdUTP/FITC-anti-BrdUTP reagents were substituted with a FITC-dUTP system, the TUNEL-positivity increased significantly, indicating that the reagents of the former kit were responsible for the lower staining efficiency.

When the chromatin of PI-dimmer sperm was decondensed before labeling with the indirect kit, staining intensity was also much improved, demonstrating the importance of access of the antibody into the compact chromatin of sperm. The molecular weight of antibodies is approximately 150-fold higher than that of FITC-dUTPs. Although in somatic cells the addition of the BrdUTP to the 3’-OH breaks might be more efficient than direct labeling methods [39], it represents a disadvantage when staining the extremely condense chromatin of spermatozoa.

Our results are important for future use of TUNEL in clinical practice. Indeed, laboratories relying on the use of antibody-based labeling systems heavily underestimate SDF, most particularly in infertile patients with reduced sperm motility. In fact, we show here that the lower the quality of the semen sample (higher percentage of PI-dimmer and lower percentage of motile spermatozoa), the lower the efficiency of the staining due to steric hindrance of the antibody to the chromatin of dead spermatozoa. As a consequence, the kit using BrdUTP/FITC-anti-BrdUTP as the staining fluorochrome should not be recommended as the standard method to assay DNA damage in semen [30–32]. From the 22 subjects used for the TUNEL kits comparison study attending our clinic, 73% were normozoospermic according to the 2010 WHO reference values. If a threshold for TUNEL positivity of 34% is considered [6], 37% of these subjects would be diagnosed as having high SDF using the direct labeling assay, while all would be diagnosed as normal using the indirect labeling assay. An accurate semen assessment concerning DNA fragmentation in these normozoospermic subjects would certainly lead to a different medical evaluation and advisement. Although the direct kit (using FITC-dUTP) is now used more frequently, the appropriateness for semen analysis of one or the other system was never studied before and, until today, several authors are still performing their SDF analysis with the indirect system [40–44].

The finding that the differences in the staining efficiency of the two kits are not the same for all subpopulations of spermatozoa in the semen sample but depend on their condition (live or dead) was surprising and may point towards the involvement of different mechanisms causing sperm death. Indeed, when TUNEL was performed together with the LIVE/DEAD staining and the results were compared in living spermatozoa only, the differences between the two labeling systems disappeared (Fig 6A). These results demonstrate that the normal chromatin condensation in healthy spermatozoa was not responsible for the differences between both kits. Since the PI-dimmer population was more affected by the steric hindrance of the antibody (Figs 3B and 6C), we hypothesized that the chromatin of this population must be compacted even more than that of dead PI-brighter spermatozoa.

Therefore, we went on to analyze more in detail the characteristics of these two dead sperm populations. By selecting only either PI-dimmer or PI-brighter populations (Fig 2C), and quantifying their TUNEL-positivity in LIVE/DEAD/FITC plots (Fig 2D and 2E upper quadrants) we were able to compare the efficiency of TUNEL staining in these two dead populations separately (Fig 6C). Although the differences between the two labeling systems were less pronounced in the dead PI-brighter spermatozoa, they remained significant. In addition, through decondensation of the DNA of PI-brighter spermatozoa, TUNEL staining became more intensive, suggesting that the steric hindrance, albeit not so extreme as in the PI-dimmer population, was also present in the dead PI-brighter spermatozoa.

We have not a clear explanation for the different condensation status of dead DNA fragmented PI- brighter and PI-dimmer spermatozoa. It is well known that condensation reflects the process of chromatin maturation and that there is a strict association between the latter and SDF. Indeed many studies have reported that sperm with DNA damage may also show poor chromatin maturation [25, 45, 46, 47], assessed as aniline blue [48] and chromomicin A staining [49] or as poor hyaluronic acid binding capacity [50]. Some studies also reported a link between sperm DNA fragmentation and the presence of nuclear large vacuoles, another sign of poor chromatin condensation [51]. Consistently, it has been proposed that sperm DNA fragmentation could derive from defects in the sperm maturation process, hampering the repair of the physiological nicks occurring to favor the replacement of the histones with protamines [52, 53]. On the other hand, the failure of reaching a proper chromatin condensation can make sperm nuclei more vulnerable to ROS (reactive oxygen species), able to break the backbone of DNA. In our system the trait of chromatin condensation is more associated to dead cells than to viable ones, indicating that it likely represents a degenerative aspect instead of a maturity trait.

Since chromatin condensation is a process occurring also during germ cell apoptosis [45] and apoptosis seems to be the main cause of SDF in both PI-dimmer and PI-brighter sperm populations [20, 25], we could speculate that DNA fragmented dead PI-brighter and PI-dimmer spermatozoa reflect, respectively, an earlier and a later stage of the apoptotic chromatin condensation.

We therefore hypothesize that the dead PI-brighter spermatozoa might consist of apoptotic spermatozoa but with yet incomplete apoptotic chromatin condensation representing earlier stages of the apoptotic program [25]. Because of this intermediate level of chromatin condensation, these spermatozoa stain with PI similarly as the brighter population, but less with the TUNEL antibody-based indirect labeling. Regarding the PI-dimmer spermatozoa, they might be cells that reached the final step of the apoptotic chromatin condensation process, making the nuclei poorly accessible to the dyes and thus explaining the lower nuclear staining of the PI dimmer spermatozoa. The lower nuclear staining of PI dimmer spermatozoa cannot be due to the loss of apoptotic DNA fragments as previously hypothesized [20]. Indeed, the present study showed that after chromatin decondensation the PI-dimmer population stained as intensively as the PI-brighter population, suggesting similar amounts of DNA in both populations.

In summary, by comparing the two kits with distinct labeling characteristics we observed several differences in the intensity of chromatin staining, ranging from similar labeling intensity in the living spermatozoa to a drastic underestimation of DNA damage in PI-dimmer spermatozoa. An underestimation of TUNEL results due to DNA compaction was first described by Mitchell et al [16], and confirmed by others [25]. In our study, we now demonstrate that, when using a large molecule for the labeling in TUNEL, the level of DNA fragmentation may be underestimated, depending on the condition of the chromatin of the sperm sample.

In other studies, the size of the subpopulation containing PI-dimmer spermatozoa has been shown to correlate with the results of conventional semen analysis, whereas the size of the subpopulation containing PI-brighter spermatozoa displaying SDF predicts male fertility irrespective the results given by conventional semen analysis [6, 24]. Our work now helps to clarify the characteristics of the PI-dimmer sperm population and points towards the presence of various sperm populations with different degrees of chromatin compaction. Further studies are needed to verify whether the relative sizes of these subpopulations may correlate with infertility.

## Supporting information

S1 Table Semen characteristics of each sample used in the study and respective assays performed. The non-normozoospermic men (2010 WHO reference values) are highlighted in bold.
(DOCX)

S2 Table Examples of commercially available kits containing the same labelling components as the ones compared in this work: BrdUTP/labelled-anti-BrdUTP and FITC-dUTP. (DOCX)

## References

[1] Singh NP, Danner DB, Tice RR, McCoy MT, Collins GD, Schneider EL. Abundant alkali-sensitive sites in DNA of human and mouse sperm. Exp Cell Res. 1989;184(2):461–70. 2806399 10.1016/0014-4827(89)90344-3

[2] Gorczyca W, Traganos F, Jesionowska H, Darzynkiewicz Z. Presence of DNA strand breaks and increased sensitivity of DNA in situ to denaturation in abnormal human sperm cells—analogy to apoptosis of somatic cells. Exp Cell Res. 1993;207(1):202–5. doi: 10.1006/excr.1993.1182 8391465 10.1006/excr.1993.1182

[3] Bungum M, Humaidan P, Spano M, Jepson K, Bungum L, Giwercman A. The predictive value of sperm chromatin structure assay (SCSA) parameters for the outcome of intrauterine insemination, IVF and ICSI. Hum Reprod. 2004;19(6):1401–8. doi: 10.1093/humrep/deh280 15117894 10.1093/humrep/deh280

[4] Duran EH, Morshedi M, Taylor S, Oehninger S. Sperm DNA quality predicts intrauterine insemination outcome: a prospective cohort study. Hum Reprod. 2002;17(12):3122–8. 12456611 10.1093/humrep/17.12.3122

[5] Morris ID, Ilott S, Dixon L, Brison DR. The spectrum of DNA damage in human sperm assessed by single cell gel electrophoresis (Comet assay) and its relationship to fertilization and embryo development. Hum Reprod. 2002;17(4):990–8. 11925396 10.1093/humrep/17.4.990

[6] Muratori M, Marchiani S, Tamburrino L, Cambi M, Lotti F, Natali I, et al DNA fragmentation in brighter sperm predicts male fertility independently from age and semen parameters. Fertil Steril. 2015;104(3):582–90. doi: 10.1016/j.fertnstert.2015.06.005 26151619 10.1016/j.fertnstert.2015.06.005

[7] Osman A, Alsomait H, Seshadri S, El-Toukhy T, Khalaf Y. The effect of sperm DNA fragmentation on live birth rate after IVF or ICSI: a systematic review and meta-analysis. Reprod Biomed Online. 2015;30:120–7. doi: 10.1016/j.rbmo.2014.10.018 25530036 10.1016/j.rbmo.2014.10.018

[8] Robinson L, Gallos ID, Conner SJ, Rajkhowa M, Miller D, Lewis S, et al The effect of sperm DNA fragmentation on miscarriage rates: a systematic review and meta-analysis. Hum Reprod. 2012;27(10):2908–17. doi: 10.1093/humrep/des261 22791753 10.1093/humrep/des261

[9] Zini A, Jamal W, Cowan L, Al-Hathal N. Is sperm dna damage associated with IVF embryo quality? A systematic review. J Assist Reprod Genet. 2011;28(5):391–7. doi: 10.1007/s10815-011-9544-6 21327499 10.1007/s10815-011-9544-6 PMC3151360

[10] Giwercman A, Lindstedt L, Larsson M, Bungum M, Spano M, Levine RJ, et al Sperm chromatin structure assay as an independent predictor of fertility in vivo: a case-control study. Int J Androl. 2010;33(1):E221–E7. doi: 10.1111/j.1365-2605.2009.00995.x 19840147 10.1111/j.1365-2605.2009.00995.x

[11] Zidi-Jrah I, Hajlaoui A, Mougou-Zerelli S, Kammoun M, Meniaoui I, Sallem A, et al Relationship between sperm aneuploidy, sperm DNA integrity, chromatin packaging, traditional semen parameters, and recurrent pregnancy loss. Fertil Steril. 2016;105(1):58–64. doi: 10.1016/j.fertnstert.2015.09.041 26493117 10.1016/j.fertnstert.2015.09.041

[12] Pfeifer S, Goldberg J, Lobo R, Thomas M, Pisarska M, Widra E, et al The clinical utility of sperm DNA integrity testing: a guideline. Fertil Steril. 2013;99(3):673–7. doi: 10.1016/j.fertnstert.2012.12.049 23391408 10.1016/j.fertnstert.2012.12.049

[13] Barratt CLR, Aitken RJ, Bjorndahl L, Carrell DT, de Boer P, Kvist U, et al Sperm DNA: organization, protection and vulnerability: from basic science to clinical applications-a position report. Hum Reprod. 2010;25(4):824–38. doi: 10.1093/humrep/dep465 20139429 10.1093/humrep/dep465

[14] Tomlinson M, Lewis S, Morroll D. Sperm quality and its relationship to natural and assisted conception: British Fertility Society Guidelines for practice. Hum Fertil (Camb). 2013;16(3):175–93. 23862664 10.3109/14647273.2013.807522

[15] World Health Organization. WHO Laboratory Manual for the Examination and Processing of Human Semen. ed t, editor. 5th ed Geneva: World Health Organization; 2010.

[16] Mitchell LA, De Iuliis GN, Aitken RJ. The TUNEL assay consistently underestimates DNA damage in human spermatozoa and is influenced by DNA compaction and cell vitality: development of an improved methodology. Int J Androl. 2011;34(1):2–13. doi: 10.1111/j.1365-2605.2009.01042.x 20158539 10.1111/j.1365-2605.2009.01042.x

[17] Evenson DP. Sperm chromatin structure assay (SCSA). Methods in molecular biology (Clifton, NJ). 2013;927:147–64. 10.1007/978-1-62703-038-0_14 22992911

[18] Fernandez JL, Muriel L, Rivero MT, Goyanes V, Vazquez R, Alvarez JG. The sperm chromatin dispersion test: A simple method for the determination of sperm DNA fragmentation. J Androl. 2003;24(1):59–66. 12514084

[19] Simon L, Lutton D, McManus J, Lewis SEM. Sperm DNA damage measured by the alkaline Comet assay as an independent predictor of male infertility and in vitro fertilization success. Fertil Steril. 2011;95(2):652–7. doi: 10.1016/j.fertnstert.2010.08.019 20864101 10.1016/j.fertnstert.2010.08.019

[20] Marchiani S, Tamburrino L, Olivito B, Betti L, Azzari C, Forti G, et al Characterization and sorting of flow cytometric populations in human semen. Andrology. 2014;2(3):394–401. doi: 10.1111/j.2047-2927.2014.00208.x 24700807 10.1111/j.2047-2927.2014.00208.x

[21] Marchiani S, Tamburrino L, Maoggi A, Vannelli GB, Forti G, Baldi E, et al Characterization of M540 bodies in human semen: Evidence that they are apoptotic bodies. Mol Hum Reprod. 2007;13(9):621–31. doi: 10.1093/molehr/gam046 17584827 10.1093/molehr/gam046

[22] Muratori M, Marchiani S, Forti G, Baldi E. Sperm ubiquitination positively correlates to normal morphology in human semen. Hum Reprod. 2005;20(4):1035–43. doi: 10.1093/humrep/deh678 15705629 10.1093/humrep/deh678

[23] Muratori M, Porazzi I, Luconi M, Marchiani S, Forti G, Baldi E. Annexin V binding and merocyanine staining fail to detect human sperm capacitation. J Androl. 2004;25(5):797–810. 15292113 10.1002/j.1939-4640.2004.tb02858.x

[24] Muratori M, Marchiani S, Tamburrino L, Tocci V, Failli P, Forti G, et al Nuclear staining identifies two populations of human sperm with different DNA fragmentation extent and relationship with semen parameters. Hum Reprod. 2008;23(5):1035–43. doi: 10.1093/humrep/den058 18326515 10.1093/humrep/den058

[25] Muratori M, Tamburrino L, Marchiani S, Cambi M, Olivito B, Azzari C, et al Investigation on the origin of sperm DNA fragmentation: role of apoptosis, immaturity and oxidative stress. Mol Med. 2015;21:109–22. doi: 10.2119/molmed.2014.00158 25786204 10.2119/molmed.2014.00158 PMC4461587

[26] Sergerie M, Laforest G, Bujan L, Bissonnette F, Bleau G. Sperm DNA fragmentation: threshold value in male fertility. Hum Reprod. 2005;20(12):3446–51. doi: 10.1093/humrep/dei231 16085665 10.1093/humrep/dei231

[27] Sharma RK, Sabanegh E, Mahfouz R, Gupta S, Thiyagarajan A, Agarwal A. TUNEL as a Test for Sperm DNA Damage in the Evaluation of Male Infertility. Urology. 2010;76(6):1380–6. doi: 10.1016/j.urology.2010.04.036 20573380 10.1016/j.urology.2010.04.036

[28] Dominguez-Fandos D, Camejo MI, Ballesca JL, Oliva R. Human sperm DNA fragmentation: Correlation of TUNEL results as assessed by flow cytometry and optical microscopy. Cytometry Part A. 2007;71A(12):1011–8. 10.1002/cyto.a.20484 17972316

[29] Sepaniak S, Forges T, Gerard H, Foliguet B, Bene MC, Monnier-Barbarino P. The influence of cigarette smoking on human sperm quality and DNA fragmentation. Toxicology. 2006;223(1–2):54–60. doi: 10.1016/j.tox.2006.03.001 16621218 10.1016/j.tox.2006.03.001

[30] Pryor WA. Bio-Assays for Oxidative Stress Status. Pryor W, editor: Elsevier; 2001.

[31] Talwar P. Manual of Assisted Reproductive Technologies and Clinical Embryology. Talwar P, editor: Jaypee Brothers Medical Publishers (P) Ltd; 2012.

[32] Zini A, Agarwal Ae. Sperm Chromatin: Biological and Clinical Applications in Male Infertility and Assisted Reproduction. Zini A, Agarwal A, editors2011.

[33] Li X, Darzynkiewicz Z. Labelling DNA strand breaks with BrdUTP. Detection of apoptosis and cell proliferation. Cell Prolif. 1995;28(11):571–9. 8555370 10.1111/j.1365-2184.1995.tb00045.x

[34] Darzynkiewicz Z, Zhao H. Detection of DNA Strand Breaks in Apoptotic Cells by Flow- and Image-Cytometry In: Didenko VV, editor. DNA Damage Detection in Situ, Ex Vivo, and in Vivo: Methods and Protocols. Methods Mol Biol. 682 2011 p. 91–101. 10.1007/978-1-60327-409-8_8 PMC3003436 21057923

[35] Muratori M, Tamburrino L, Marchiani S, Guido C, Forti G, Baldi E. Critical aspects of detection of sperm DNA fragmentation by Tunel/Flow Cytometry. Syst Biol Reprod Med. 2010;56(4):277–85. doi: 10.3109/19396368.2010.489660 20718615 10.3109/19396368.2010.489660

[36] Antonucci N, Manes S, Corradetti B, Manicardi GC, Borini A, Bizzaro D. A novel in vitro sperm head decondensation protocol for rapid flow cytometric measurement of deoxyribonucleic acid content. Fertil Steril. 2013;99(7):1857–61. doi: 10.1016/j.fertnstert.2013.02.014 23498889 10.1016/j.fertnstert.2013.02.014

[37] Cissen M, van Wely M, Scholten I, Mansell S, de Bruin JP, Mol B, et al Measuring Sperm DNA Fragmentation and Clinical Outcomes of Medically Assisted Reproduction: A Systematic Review and Meta-Analysis. PLoS One. 2016;11(11). 10.1371/journal.pone.0165125 PMC5104467 27832085

[38] Marchiani S, Tamburrino L, Giuliano L, Nosi D, Sarli V, Gandini L, et al Sumo1-ylation of human spermatozoa and its relationship with semen quality. Int J Androl. 2011;34(6):581–93. 21039605 10.1111/j.1365-2605.2010.01118.x

[39] Darzynkiewicz Z, Li X, Traganos F, inventorsMethods for labeling DNA ends with halogenated nucleotides and detecting same with antibodies USA1999.

[40] López-Úbeda R, García-Vázquez F, Gadea J, Matás C. Oviductal epithelial cells selected boar sperm according to their functional characteristics. Asian J Androl. 2016;18:1–8. 10.4103/1008-682X.173936 PMC5507082 27232850

[41] Kumar D, Kumar P, Singh P, Yadav SP, Yadav PS. Assessment of sperm damages during different stages of cryopreservation in water buffalo by fluorescent probes. Cytotechnology. 2016;68(3):451–8. doi: 10.1007/s10616-014-9798-9 25373338 10.1007/s10616-014-9798-9 PMC4846636

[42] Park L, Neri QV, Rosenwaks Z, Palermo GD. Seminal fluid ROS-buffering capacity relates to sperm parameters, chromatin integrity and embryo developmental competence. Hum Reprod. 2015;30:147-.

[43] Del Olmo E, Bisbal A, Garcia-Alvarez O, Maroto-Morales A, Ramon M, Jimenez-Rabadan P, et al Free-radical production after post- thaw incubation of ram spermatozoa is related to decreased in vivo fertility. Reprod Fert Develop. 2015;27(8):1187–96. 10.1071/RD14043 25485567

[44] Bolanos JMG, da Silva CMB, Munoz PM, Rodriguez AM, Davila MP, Rodriguez-Martinez H, et al Phosphorylated AKT preserves stallion sperm viability and motility by inhibiting caspases 3 and 7. Reproduction. 2014;148(2):221–35. doi: 10.1530/REP-13-0191 24850868 10.1530/REP-13-0191

[45] Baccetti B, Collodel G, Piomboni P. Apoptosis in human ejaculated sperm cells (Notulae seminologicae 9). J Submicrosc Cytol Pathol. 1996;28(4):587–96. 8933742

[46] Sakkas D, Seli E, Bizzaro D, Tarozzi N, Manicardi GC. Abnormal spermatozoa in the ejaculate: abortive apoptosis and faulty nuclear remodelling during spermatogenesis. Reprod Biomed Online. 2003;7(4):428–32. 14656403 10.1016/s1472-6483(10)61886-x

[47] Oliva R. Protamines and male infertility. Hum Reprod Update. 2006;12(4):417–35. doi: 10.1093/humupd/dml009 16581810 10.1093/humupd/dml009

[48] Sati L, Ovari L, Bennett D, Simon SD, Demir R, Huszar G. Double probing of human spermatozoa for persistent histones, surplus cytoplasm, apoptosis and DNA fragmentation. Reprod Biomed Online. 2008;16(4):570–9. 18413066 10.1016/s1472-6483(10)60464-6

[49] De Iuliis GN, Thomson LK, Mitchell LA, Finnie JM, Koppers AJ, Hedges A, et al DNA Damage in Human Spermatozoa Is Highly Correlated with the Efficiency of Chromatin Remodeling and the Formation of 8-Hydroxy-2 '-Deoxyguanosine, a Marker of Oxidative Stress. Biol Reprod. 2009;81(3):517–24. doi: 10.1095/biolreprod.109.076836 19494251 10.1095/biolreprod.109.076836

[50] Tarozzi N, Nadalini M, Bizzaro D, Serrao L, Fava L, Scaravelli G, et al Sperm-hyaluronan-binding assay: clinical value in conventional IVF under Italian law. Reprod Biomed Online. 2009;19:35–43. 20034422 10.1016/s1472-6483(10)60282-9

[51] Boitrelle F, Guthauser B, Alter L, Bailly M, Wainer R, Vialard F, et al The nature of human sperm head vacuoles: a systematic literature review. Basic Clin Androl. 2013;29(23:3). doi: 10.1186/2051-4190-23-3 25780567 10.1186/2051-4190-23-3 PMC4346294

[52] Marcon L, Boissonneault G. Transient DNA strand breaks during mouse and human spermiogenesis: New insights in stage specificity and link to chromatin remodeling. Biol Reprod. 2004;70(4):910–8. doi: 10.1095/biolreprod.103.022541 14645105 10.1095/biolreprod.103.022541

[53] Sakkas D, Manicardi G, Bianchi PG, Bizzaro D, Bianchi U. Relationship between the presence of endogenous nicks and sperm chromatin packaging in maturing and fertilizing mouse spermatozoa. Biol Reprod. 1995;52(5):1149–55. 7626715 10.1095/biolreprod52.5.1149

