# Supplementary material for: TUNEL labeling with BrdUTP/anti-BrdUTP greatly underestimates the level of sperm DNA fragmentation in semen evaluation
Source: PLoS One. 2017 Aug 7;12(8):e0181802. doi: 10.1371/journal.pone.0181802 (PMC5546573; doi:10.1371/journal.pone.0181802)
Supplement: S1 Table — The non-normozoospermic men (2010 WHO reference values) are highlighted in bold. (DOCX) [file pone.0181802.s001.docx]

**S1 Table: Semen characteristics of each sample used in the study and respective assays performed.** The non-normozoospermic samples and abnormal semen values (concentration <15 x10^6^ /ml, progressive motility <32%, normal forms <4% or immotility >60%) (2010 WHO reference values) are highlighted in bold. Progressive motility refers to spermatozoa that are moving actively, either linearly or in large circles, (non-progressive motility refers to sperm that move without forward progression). Immotility refers to spermatozoa without any movement.

| Sample | Volume (ml) | Concentration  (x 10^6^/ml) | Progressive motility (%) | Immotility (%) | Normal forms (%) | Assay |
| --- | --- | --- | --- | --- | --- | --- |
| 1 | 6.5 | 87 | 54 | 38 | 7.7 | TUNEL |
| **2** | 2.6 | 106 | **29** | **64** | 9.1 | TUNEL |
| 3 | 4.9 | 30 | 47 | 46 | 6.5 | TUNEL |
| 4 | 4.3 | 28.2 | 47 | 45 | 4.8 | TUNEL |
| 5 | 4.2 | 37 | 41 | 54 | 11.2 | TUNEL |
| 6 | 2.3 | 77 | 70 | 24 | 14.4 | TUNEL & TUNEL-LIVE/DEAD |
| 7 | 2.1 | 29 | 46 | 48 | 6 | TUNEL & TUNEL-LIVE/DEAD |
| **8** | 4.1 | 62 | 43 | 57 | **3.2** | TUNEL |
| 9 | 1.8 | 82 | 32 | 50 | 6.2 | TUNEL & TUNEL-LIVE/DEAD |
| 10 | 5.8 | 50 | 45 | 43 | 6.9 | TUNEL &TUNEL-LIVE/DEAD |
| 11 | 3.6 | 125 | 52 | 35 | 10.4 | TUNEL & TUNEL-LIVE/DEAD |
| **12** | 3.8 | 16 | 39 | 50 | **1.9** | TUNEL& Kits components exchange |
| 13 | 5.5 | 24 | 36 | 53 | 8.8 | TUNEL & Kits components exchange |
| 14 | 6.1 | 101 | 64 | 25 | 13.2 | TUNEL & Kits components exchange |
| 15 | 6.1 | 20 | 48 | 49 | 13.1 | TUNEL |
| 16 | 2.8 | 35 | 41 | 54 | 6.1 | TUNEL |
| **17** | 1.6 | 212 | **23** | **70** | 7.2 | TUNEL |
| **18** | 4 | 20 | 32 | **68** | 6.2 | TUNEL & TUNEL-LIVE/DEAD |
| 19 | 2.5 | 101 | 48 | 51 | 5.2 | TUNEL & TUNEL-LIVE/DEAD |
| **20** | 4.2 | 8 | **21** | **77** | 7.5 | TUNEL |
| 21 | 4 | 100 | 48 | 45 | 4 | TUNEL & TUNEL-LIVE/DEAD |
| 22 | 4.1 | 24 | 48 | 43 | 6.5 | TUNEL & TUNEL-LIVE/DEAD |
| 23 | 2 | 30 | 34 | 56 | 6 | Kits components exchange |
| 24 | 4.8 | 30 | 66 | 28 | 5.4 | Kits components exchange |
| **25** | 4.8 | 34 | **17** | **74** | 5.4 | TUNEL-LIVE/DEAD |
| 26 | 6.6 | 17 | 72 | 26 | 4 | Decondensation tests |
| 27 | 6.3 | 65 | 47 | 46 | 15.5 | Decondensation tests |
| 28 | 3.7 | 56 | 43 | 48 | 4.9 | Decondensation/Sorting/TUNEL |
| **29** | 2.8 | 46 | **27** | **67** | 10.5 | Decondensation/Sorting/TUNEL |
| **30** | 3.7 | 15 | 57 | 41 | **3.3** | Decondensation/Sorting/TUNEL |
| **31** | 2.5 | 40 | **29** | **65** | 5 | Decondensation/Sorting/TUNEL |
| 32 | 4.9 | 56.3 | 52 | 43 | 11.5 | Decondensation/Sorting/TUNEL |
