# Supplementary material for: TUNEL labeling with BrdUTP/anti-BrdUTP greatly underestimates the level of sperm DNA fragmentation in semen evaluation
Source: PLoS One. 2017 Aug 7;12(8):e0181802. doi: 10.1371/journal.pone.0181802 (PMC5546573; doi:10.1371/journal.pone.0181802)
Supplement: S2 Table — (DOCX) [file pone.0181802.s002.docx]

**S2 Table: Examples of commercially available kits containing the same labelling components as the ones compared in this work: BrdUTP/labelled-anti-BrdUTP and FITC-dUTP.**

| **TUNEL labelling system** | |
| --- | --- |
| **BrdUTP/labelled-anti-BrdUTP** | **FITC-dUTP** |
| Apo BrdU In situ DNA fragmentation kit (BioVision) | In Situ Cell Death Detection Kit  (Roche) |
| Apo Target Apo-BrdU TUNEL assay kit  (Life technologies) | APO-DIRECT™ Kit  (BD Pharrmigen) |
| Apo-Brdu  (Calbiochem) | TUNEL Apo-Green Detection Kit  (Biotool) |
| Apo-BrdU flow cytometry kit  (BIO-RAD) | TUNEL Two-Color Apoptosis Detection Kit  (GenScript) |
| Apo-BrdU kit  (BD Pharmigen) | Apo Direct TUNEL kit  (Phoenix Flow Systems) |
| Apo-BrdU assay  (Phoenix Flow systems) | DeadEnd™ Fluorometric TUNEL System  (Promega) |
| In situ BrdU DNA Fragmentation Assay Kit (Abnova) | In situ direct DNA fragmentation (TUNEL) Assay  (Abcam) |
| Apo-BrdU kit  (ENZO) | [In Situ Cell Death Detection Kit](http://www.sigmaaldrich.com/catalog/product/roche/11684795910)  [(Sigma-Aldrich](http://www.sigmaaldrich.com/catalog/product/roche/11684795910)) |
| Apo-BrdU kit  (Tonbo Biosciences) | TUNEL FITC Apoptosis Detection Kit  (Vazyme Biotech) |
| proTUNEL DNA fragmentation assay kit  (GeneTex) | MEBSTAIN Apoptosis Kit Direct  (MBL Life Science) |
| APO-BRDU (TUNEL) Apoptosis Kit  (NOVUS Biologics) | TUNEL BrightGreen Apoptosis Detection Kit  (Green Mountain Biosystems) |
